# Periscope of Dermatology

**Published:** 1897-05-15

**Authors:** 


### PERISCOPE OF DERMATOLOGY.

**Leprosy.**—Grossmann[1] has investigated the occurrence of leprosy in Europe, and considers that the disease is not on the decrease, except, perhaps, in Norway, where, within a comparatively short time, the number of lepers has been reduced from three thousand to eight hundred, owing to isolation or similar measures. In Iceland, although he found a considerable number of lepers, an astonishing indifference seems to reign towards this terrible disease; thus, it is the custom of the country to kiss all those present, even if they may have leprous ulcerations all over the face; with regard to the etiology, he considers the disease is produced by the specific bacillus; and is transmitted by contact. He does not consider that heredity is responsible for any cases. Since no successful remedy for the disease has been found, our main duty must be to isolate infectious cases as much as possible.

The *Lancet*[2] comments on the discovery of leprosy in the district of Memel, in Eastern Prussia. The Government has determined on the erection of a special hospital in this district, in consequence of the representations of Dr. Koch, who also insists on the registration and notification of all cases, and compulsory isolation in special hospitals; besides which, a close watch is to be kept on the adjoining Russian territory, from which the disease has been introduced.

Morrow[3] condemns the custom in New York of confining the lepers for life in a hospital outside the city. He believes that if a leper provides himself with a separate room, bed, and board, he is practically innocuous, so far as contagion is concerned; if he requires medical care, he can be treated in special wards of the ordinary hospitals. He points out that if these stringent regulations, now in force for the isolation of lepers, are carried out, similar isolation should be provided for tubercular patients, since during the last ten years over sixty thousand deaths have been recorded from tuberculosis, while within the same period only two deaths were recorded from leprosy.

Ashmead[4] strongly urges the isolation of lepers, and quotes a large number of authorities who support this view.

Joseph[5] thinks that there is no difficulty in diagnosing a case of leprosy, since its nature is apparent at first glance. He is strongly in favour of isolation, and wishes that they should all be made to leave Germany, unless there is an international law against it.

Carasquilla[6] states that he has treated one hundred cases of leprosy by serum from horses, which he had immunised against the disease, by injecting 15 to 60 cubic centimetres of leprous serum every two days on three occasions. The serum taken with great care is then employed as follows: In a leper from whom blood to the amount of 150 to 250 cubic centimetres has been drawn, 1 to 5 cubic centimetres of horse's serum is injected; a second injection is made three or four days later, according to the degree of reaction; then a third and a fourth; in some subjects reaction does not take place till after this period. The reaction manifests itself, as in the case of other serums, by fever, circulatory disturbances, changes in the secretions, &c. After some days the leprous lesions undergo somewhat rapid modification; the tubercles desquamate and shrink, the ulcerations become vegetating and cicatrice. The disturbances of sensibility are lessened, and, when the cases are not too far advanced, improvement takes place rapidly and to an astonishing degree.

Gallet[7] treated four cases of leprosy with methylene blue hypodermically; in two cases the same drug was given by the mouth; the results were nil. He also treated cases with asses' serum, and considered the results were encouraging.

Bisnier[8] regards a long course of electro-galvanic cauterisation as a certain means of reducing leprous tubercular formation, wherever localised. Each tubercle is cauterised instentitially by single or multiple points, after which daily spraying with carbolic acid and dressing with sublimate gauze should be employed. The galvano cautery should also be employed to the mucous membranes.

[1] Brit Med. Journ., Dec. 5, 1896, p. 1,631. [2] Lancet, Dec. 5, 1896, p. 1,636. [3] New York Med. Rec., Nov. 7, 1896, p. 615. [4] Ditto, Jan. 23, 1897, p. 142. [5] Berl. Klin. Woch, 1896, No. 37. Central. fur. Chirurgic., No. 5, p. 132. [6] Brit. Med. Journ. Ep., Dec. 19, 1896; Med. Week, Sept. 4, 1896, p. 431. [7] Brit. Journ. of Derm., Oct., 1897, p. 87. [8] Am. Journ. of Med. Sci., Feb., 1897, p. 232.

